# A rare case of purulent massive pericardial effusion in an 85- years-old male: case report

**DOI:** 10.1017/ash.2025.89

**Published:** 2025-09-03

**Authors:** Ira Vori, Setyasih Anjarwani, Cholid Tri Tjahjono

**Affiliations:** 1 Resident of Cardiology and Vascular Department, Faculty of MedicineUniversitas Brawijaya Malang, Saiful Anwar General Hospital East Java; 2 Staff of Cardiology and Vascular Department, Faculty of MedicineUniversitas Brawijaya Malang, Saiful Anwar General Hospital East Java

**Keywords:** Bacterial Pericarditis, Secondary pericarditis, Pericardial effusion, Purulent pericarditis, Pneumonia, Pericardiocentesis

## Abstract

**Introduction:** Purulent pericarditis is defined as an infection in the pericardial space that produces macroscopically or microscopically purulent fluid. It was a rare but life-threatening condition. It may be primary or secondary to another infectious process. The diagnosis can only be confirmed by pericardiocentesis. Treatment must include drainage of the pericardial space combined with systemic antibiotics. This case report focuses on a critical and rare clinical scenario of purulent massive pericardial effusion in an 85- year-old male patient. This condition, characterized by an infectious or inflammatory accumulation of fluid in the pericardial cavity, presents significant diagnostic and therapeutic challenges, particularly in the context of multiple comorbidities. **Case Description:** The patient’s presentation, complicated by pneumonia, diabetes mellitus (DM), and heart failure, underscores the complexities in diagnosing and managing elderly patients with diverse medical backgrounds. The diagnosis of massive pericardial effusion was confirmed through echocardiography, which revealed the purulent fluid from pericardiocentesis procedure, a finding critical for guiding the diagnostic and management strategy. The source of infection wasn’t clear in patient with immunocompromised condition. Some examination performed to find the source of infection that led to a subdiaphragmatic suppurative focus. Infection management was good, but the patient ended with a constrictive that make his condition worse. The patient passed away on the 10^th^ day of hospitalization. **Conclusion:** It is importance to recognize and promptly address purulent massive pericardial effusion in elderly patients with complex medical histories. The successful clinical outcome following the pericardiocentesis and the adaptive antimicrobial treatment approach provides valuable insights into the management of this severe condition.

